# Eptifibatide-induced acute profound thrombocytopenia: A case report

**DOI:** 10.1097/MD.0000000000028243

**Published:** 2022-10-21

**Authors:** Mohammed A. Alamin, Abdulrahman Al-Mashdali, Dawoud I. Al Kindi, Elkhansa A. Elshaikh, Fahmi Othman

**Affiliations:** a Hamad General Hospital, Internal Medicine Department, Hamad Medical Corporation, Doha, Qatar; b Department of Adult Cardiology, Heart Hospital, Hamad Medical Corporation, Doha, Qatar; c Othman Digna Hospital, Port-Sudan, Sudan.

**Keywords:** acute coronary syndrome, drug-induced thrombocytopenia, eptifibatide, glycoprotein IIa/IIIb inhibitors, thrombocytopenia

## Abstract

Eptifibatide is an antiplatelet agent used in the medical management of acute coronary syndrome. Although multiple studies did not reveal a significant association between eptifibatide and the development of thrombocytopenia, recent case reports brought attention to this relatively rare side effect.

**Patient concerns:**

We report a 61 years old male with acute coronary syndrome who underwent primary coronary intervention.

**Diagnosis and intervention:**

The patient developed acute profound thrombocytopenia following eptifibatide administration. Following prompt offending drug discontinuation, the platelet counts recovered, without clinical sequelae or the need for platelet transfusion. Dual antiplatelet therapy with aspirin and clopidogrel was resumed after platelet count normalization.

**Outcomes:**

The patient had a normal platelet count and no bleeding events on follow-up after three months upon discharge.

**Conclusion:**

Eptifibatide, a glycoprotein IIa/IIIb inhibitor used in the management of acute coronary syndrome, can induce acute, profound thrombocytopenia that can have significant morbidity in patients. This case highlights this relatively rare side effect and the importance of monitoring blood counts and observing for any signs of bleeding or thrombosis that might occur in such patients.

## 1. Introduction

Eptifibatide is an antiplatelet agent that reversibly inhibits the glycoprotein IIb/IIIa receptors. Several trials demonstrated its efficacy in the management of acute coronary syndrome (ACS) undergoing percutaneous coronary intervention.^[1–5]^ The major adverse effect is bleeding,^[1]^ although thrombocytopenia is increasingly reported.^[6,7]^ The incidence of eptifibatide-induced thrombocytopenia is extremely low (0.2%).^[1,3]^ The platelet counts recover promptly following drug discontinuation and rarely require platelet transfusion.^[7]^ We present a case of profound thrombocytopenia following eptifibatide administration in a 61 years old male admitted with ACS.

## 2. Case presentation

A 61 years old male with a past medical history of type 2 diabetes mellitus and hypertension presented to the emergency department with severe epigastric pain radiating to the back, shoulders, and both arms. He had no previous history of thrombocytopenia, hematology disorders, or cardiovascular diseases. He also denied any previous exposure to heparin or eptifibatide.

Clinical examination revealed stable hemodynamics and normal cardiovascular findings. His blood pressure was 133/70 mm Hg and equal in all limbs. Electrocardiography showed T wave inversion in the lateral leads (I, avL, V4-V6). Serum troponins were positive. The patient was admitted as a case of non-ST-segment elevation myocardial infarction elevation myocardial infarction; aortic dissection was excluded with computed tomography scan of the aorta. Patient hemoglobin on admission was 16.7 mg/dL; platelet count was 197,000/μL; renal and liver parameters were within a normal range.

**Figure 1.**
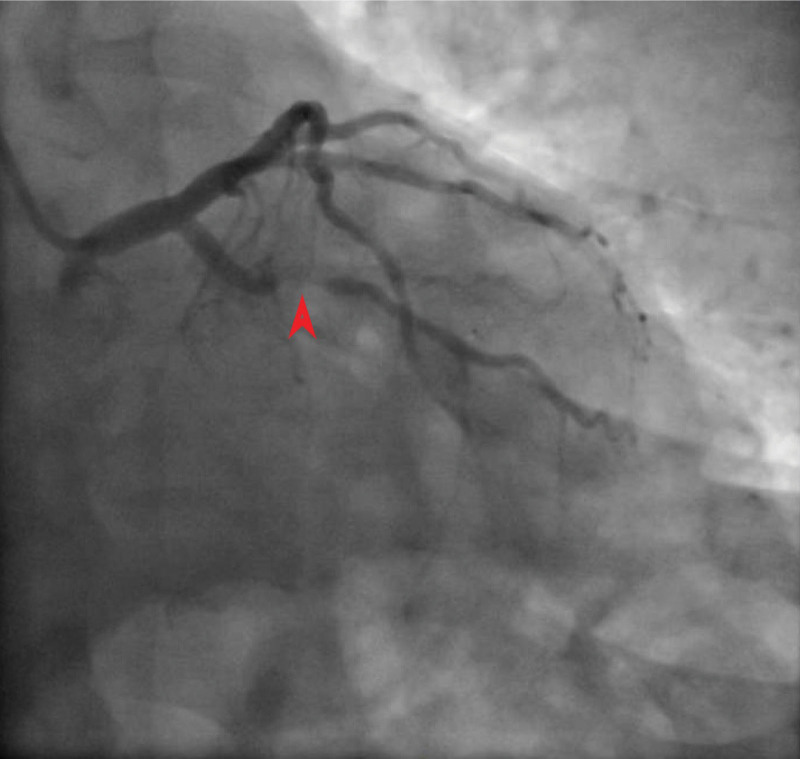
Coronary angiography image demonstrating (red arrow-head) 99% stenosis at the obtuse marginal artery (OM1).

The patient was treated with aspirin, clopidogrel, atorvastatin, and enoxaparin, along with amlodipine and insulin for hypertension and diabetes control. Coronary angiography revealed 99% stenosis of the first obtuse marginal artery, the lesion was stented with a drug-eluting stent, and the patient started on eptifibatide 180 mcg/kg intravenous infusion for 18 hours (Fig. 1). He had mild bleeding from the radial access site 3 hours after the procedure that stopped with pressure; otherwise, no major bleeding was documented. The next day, a complete blood count revealed a platelet count of 8000/μL (before angiography, platelets were 176,000/μL). Upon clinical assessment, the patient was hemodynamically stable, without any evidence of bleeding, ecchymosis, or signs of deep vein thrombosis.

**Figure 2.**
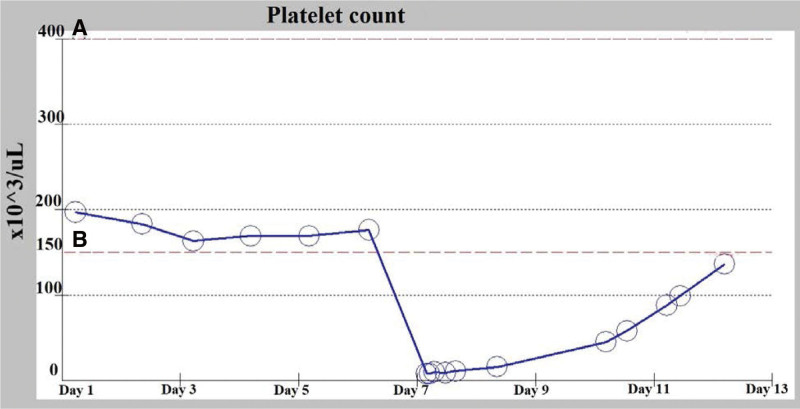
Demonstrating platelet count during hospitalization; the normal level at baseline (day 1), the acute drop occurred with eptifibatide infusion (day 7), and the recovery of the platelet count after discontinuation of the drug. (a) In the figure indicates normal high level (400 × 10^3^/μL). (b) In the figure indicates normal low level (150 × 10^3^/μL).

Aspirin and clopidogrel were suspended, heparin-induced thrombocytopenia (HIT) test was sent, and the result was negative. Repeated readings of platelets count ranged between 8 and 16/μL within the following 2 days. Forty-eight hours from the initial profound thrombocytopenia documentation, the patient’s platelets count increased to 44,000/μL; clopidogrel resumed, no bleeding or thrombotic events were noted, and the patient did not require platelets transfusion (Fig. 2).

Ninety-six hours after the initial event, platelets count increased to 99,000/μL; aspirin was resumed, the patient was observed for one more day and discharged in good clinical status. Three months later, the patient was seen in the outpatient department, was in a good clinical state with no history of bleeding; the platelet count was 204,000/μL.

## 3. Discussion

Eptifibatide is a glycoprotein IIa/IIIb (Gb IIa/IIIb) inhibitor used in the treatment of ACS.^[1–5]^ The use of Gb IIa/IIIb inhibitors has been reported to cause profound thrombocytopenia.^[6]^ Most studies categorized thrombocytopenia as mild (platelet counts < 100,000/μL), severe (platelet counts < 50,000/μL), and profound with counts < 20,000/μL).^[6]^ Abciximab, a Gb IIa/IIIb inhibitor, is known to cause thrombocytopenia. In fact, in a previous pooled analysis, abciximab, but not eptifibatide or tirofiban, increased the incidence of thrombocytopenia compared to placebo in patients who receive heparin.^[7]^ However, recently this side effect has also been reported with eptifibatide use.^[6,8,9]^ The incidence of severe and profound thrombocytopenia associated with eptifibatide was estimated to be around 0.2%.^[1,3]^ Our patient received aspirin, clopidogrel, enoxaparin, and eptifibatide; the latter 3 drugs have been associated with thrombocytopenia in the literature.^[10]^ The HIT test was done twice and was negative, which minimized the likelihood of heparin-induced thrombocytopenia; in addition, the platelets count level was extremely low for HIT.^[11]^ Although clopidogrel may rarely lead to thrombotic thrombocytopenic purpura,^[12]^ there was no clinical or biochemical evidence of this syndrome in our patient, and platelets count was not affected following reinitiation of clopidogrel.

The acute onset of the profound thrombocytopenia following eptifibatide administration, which was confirmed on a peripheral smear (excluding pseudo thrombocytopenia), the twice negative HIT test, along with the absence of features of thrombotic thrombocytopenic purpura, and the recovery of platelet later, made eptifibatide the most likely culprit agent for the event. Eptifibatide-induced acute profound thrombocytopenia can be associated in some occasions with thrombosis and disseminated intravascular coagulopathy^[9]^; fortunately, our patient did not develop any bleeding or thrombotic complications, although platelet count was repeated 18 hours after angioplasty. His platelet counts started to recover after 48 hours of eptifibatide infusion; later on, aspirin and clopidogrel were resumed, and the patient platelet count returned to normal.

## 4. Conclusion

This case adds to the current evidence that eptifibatide can lead to the occurrence of acute profound thrombocytopenia, albeit the incidence is rare. It also highlights the importance of frequent platelet count monitoring and observation for any bleeding or thrombotic events following eptifibatide initiation.

## Author contributions

**Conceptualization:** Mohammed A. Alamin.

**Supervision:** Fahmi Othman.

**Validation:** Dawoud I. Al Kindi, Elkhansa A. Elshaikh.

**Writing – original draft:** Mohammed A. Alamin, Abdulrahman Al-Mashdali.

**Writing – review & editing:** Mohammed A. Alamin, Abdulrahman Al-Mashdali, Dawoud I. Al Kindi, Elkhansa A. Elshaikh, Fahmi Othman.
